# Depth and Dissolved Organic Carbon Shape Microbial Communities in Surface Influenced but Not Ancient Saline Terrestrial Aquifers

**DOI:** 10.3389/fmicb.2018.02880

**Published:** 2018-11-27

**Authors:** Margarita Lopez-Fernandez, Mats Åström, Stefan Bertilsson, Mark Dopson

**Affiliations:** ^1^Centre for Ecology and Evolution in Microbial Model Systems, Linnaeus University, Kalmar, Sweden; ^2^Department of Biology and Environmental Science, Linnaeus University, Kalmar, Sweden; ^3^Limnology and Science for Life Laboratory, Department of Ecology and Genetics, Uppsala University, Uppsala, Sweden

**Keywords:** 16S rRNA gene, amplicon sequencing, deep subsurface, groundwaters, chemistry, microbial diversity

## Abstract

The continental deep biosphere is suggested to contain a substantial fraction of the earth’s total biomass and microorganisms inhabiting this environment likely have a substantial impact on biogeochemical cycles. However, the deep microbial community is still largely unknown and can be influenced by parameters such as temperature, pressure, water residence times, and chemistry of the waters. In this study, 21 boreholes representing a range of deep continental groundwaters from the Äspö Hard Rock Laboratory were subjected to high-throughput 16S rRNA gene sequencing to characterize how the different water types influence the microbial communities. Geochemical parameters showed the stability of the waters and allowed their classification into three groups. These were (i) waters influenced by infiltration from the Baltic Sea with a “modern marine (MM)” signature, (ii) a “thoroughly mixed (TM)” water containing groundwaters of several origins, and (iii) deep “old saline (OS)” waters. Decreasing microbial cell numbers positively correlated with depth. In addition, there was a stronger positive correlation between increased cell numbers and dissolved organic carbon for the MM compared to the OS waters. This supported that the MM waters depend on organic carbon infiltration from the Baltic Sea while the ancient saline waters were fed by “geogases” such as carbon dioxide and hydrogen. The 16S rRNA gene relative abundance of the studied groundwaters revealed different microbial community compositions. Interestingly, the TM water showed the highest dissimilarity compared to the other two water types, potentially due to the several contrasting water types contributing to this groundwater. The main identified microbial phyla in the groundwaters were Gammaproteobacteria, unclassified sequences, Campylobacterota (formerly Epsilonproteobacteria), Patescibacteria, Deltaproteobacteria, and Alphaproteobacteria. Many of these taxa are suggested to mediate ferric iron and nitrate reduction, especially in the MM waters. This indicated that nitrate reduction may be a neglected but important process in the deep continental biosphere. In addition to the high number of unclassified sequences, almost 50% of the identified phyla were archaeal or bacterial candidate phyla. The percentage of unknown and candidate phyla increased with depth, pointing to the importance and necessity of further studies to characterize deep biosphere microbial populations.

## Introduction

Despite being isolated from the photosynthetic fueled surface by time and distance, the continental subsurface is estimated to contain up to 19% of the earth’s total biomass (McMahon and Parnell, 2014). This vast subsurface community is comprised of all three domains of life plus viruses (reviewed in Itavaara et al., 2016), is at least partially active (Schippers et al., 2005; Eydal et al., 2009; Morono et al., 2011; Orsi et al., 2013), and affects global nutrient and energy dynamics including the carbon (Pedersen and Ekendahl, 1992), sulfur (Pedersen et al., 2014), and nitrogen (Nielsen et al., 2006) cycles. In addition, the activity of microorganisms inhabiting the continental deep biosphere is important for, e.g., long-term disposal of nuclear waste where sulfate reduction to sulfide can result in the corrosion of the copper canisters used to store spent fuel (Standish et al., 2016).

Life is always dependent on the presence of water but deep continental microbial communities are also constrained by boundaries including the size of the water filled rock fractures, physical parameters such as temperature and pressure that increase with depth from the surface, and the chemical constituents of the waters such as electron donors and acceptors for microbial energy conservation. Waters present in deep hard bedrock fractures are typically oligotrophic and the amount of dissolved organic carbon (DOC) decreases with the degree of separation from the surface (Magnabosco et al., 2016). This results in higher microbial diversities in waters with more rapid replenishment that are supported by organic carbon compared to older, more isolated waters where the microbial community is maintained by carbon dioxide and hydrogen of geological origin (Purkamo et al., 2015; Wu et al., 2015; Hubalek et al., 2016; Kieft et al., 2018; Lopez-Fernandez et al., 2018b). Due to the difficulty in accessing the deep continental biosphere, many studies are carried out in mines (e.g., Pfiffner et al., 2006) or boreholes (e.g., Itavaara et al., 2011), providing access to waters with a very similar geographical proximity and only a single or minimal range of origins. This results in a limited and likely incomplete picture of the true microbiological diversity in the continental deep sub-surface being presented.

One site that circumvents the problem of accessing groundwaters with a range of characteristics is the Äspö Hard Rock Laboratory (Äspö HRL) situated on the Baltic Sea coast in southeast Sweden. This underground laboratory consists of a 3.6 km long tunnel, partially extending under the Baltic Sea, which descends to a depth of 460 m below sea level (mbsl). The Äspö HRL is built in Paleoproterozoic granitoids, which contain an abundance of mainly vertical to sub-vertical fractures and fracture zones where water of different sources and ages flows (Drake et al., 2012). These aquifers include: old saline (OS) water that resides in and flows up from deep parts of the bedrock system; brackish water that has penetrated from the overlying Baltic Sea and its predecessors (e.g., the Littorina Sea); and fresh waters introduced via rain, melting snow, and retreating glaciers in the Pleistocene (Laaksoharju et al., 2008a; Mathurin et al., 2012, 2014a). Besides providing access to multiple water types, the Äspö HRL alleviates many problems associated with contamination of deep biosphere microbiological studies as the tunnel was constructed over 30 years ago and the groundwaters continually flow toward the tunnel by hydrostatic pressure (as opposed to pumping). However, how the geochemistry of the different groundwaters demarcates the deep biosphere prokaryote diversity, richness, and metabolic strategies has not been evaluated in such a large area.

Many previous investigations have described Äspö HRL microbial communities, including cultivation-based approaches (Pedersen, 2013), ATP analysis (Eydal and Pedersen, 2007), high-throughput 16S rRNA gene inventories (Hubalek et al., 2016), and viability studies (Lopez-Fernandez et al., 2018a), as well as metagenome-based reconstructions of planktonic (Wu et al., 2015) and biofilm (Wu et al., 2017) communities. In addition, a recent metatranscriptome-based study revealed that the three domains of life are active in the deep continental biosphere (Lopez-Fernandez et al., 2018b). These studies highlight a broad range of metabolic strategies including nitrate, sulfate, and sulfur reduction along with simple fermentative microbes with a very small cell size that readily pass through a 0.22 μm membrane filter that is typically used for cell capture (Wu et al., 2015). These past findings emphasize the need to carry out a systematic and broader survey of the microbial communities in all of the different Äspö HRL water types.

In this study, we have sampled 21 boreholes representing a broad range of groundwaters dominated by meteoric, Baltic Sea, and OS origins along with mixtures thereof. The sampled boreholes intersect groundwaters with an approximate underground area of 300 m × 300 m and a depth down to 1000 m making this the first large-scale three-dimensional microbiological investigation of the deep biosphere. Statistical correlations between chemical characteristics and the microbial communities based on high-throughput sequencing of the 16S rRNA gene were used to investigate how the communities are influenced by the different water types.

## Materials and Methods

### Sampling Site and Boreholes

**FIGURE 1:**
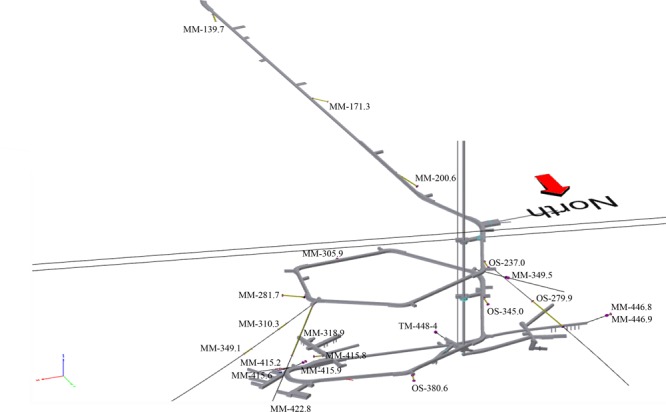
Three-dimensional model of the 21 sampled boreholes showing their position along the Äspö HRL tunnel, depth below sea level, and the water types contained therein. The borehole designations (MM, modern marine; TM, thoroughly mixed; and OS, old saline) refer to those given in Table 1.

The Swedish Nuclear Fuel and Waste Management Company (SKB)-operated Äspö HRL is situated on the Baltic Sea coast (Lat N 57° 26′ 4′′ Lon E 16° 39′ 36′′). Twenty-one boreholes (Table 1) were sampled that extend outward from the tunnel walls to cover an approximate total area of 300 × 300 × 1000 m (Figure 1).

**Table 1:** Type and chemical composition of the sampled groundwaters in May 2016 (November 2015 only for SA2074A-1).

Borehole	Designation	Water type	Depth^a^	pH^b^	δ^18^O^c^	DOC	${\mathrm{HCO}}_{3}^{–}$	${\mathrm{NH}}_{4}^{+}$	Fe^2+^	S^2-^	${\mathrm{SO}}_{4}^{2–}$	Cl^-^	${\mathrm{NO}}_{2}^{–}$	${\mathrm{NO}}_{3}^{–}$	${\mathrm{PO}}_{4}^{3–}$
SA1009B-1	MM-139.7	Marine signature (MM)	139.72	7.5	–7.3	NA^d^	189	NA	NA	0.03	124	3083	0.0004	0.0003	0.0747
SA1229A-1	MM-171.3	Marine signature (MM)	171.26	7.3	–7.3	6.4	255	5.06	1.80	0.07	90.5	3103	0.0005	BD	0.0280
SA1420A-1	MM-200.6	Marine signature (MM)	200.56	7.5	–7.5	6.5	211	1.58	0.90	0.10	104	2947	0.0005	0.0010	0.0068
SA2074A-1	MM-281.7	Marine signature (MM)	281.65	7.7	–8.2	NA	154	NA	NA	0.03	112	2854	0.0008	0.0232	0.0010
SA2273A-1	MM-305.9	Marine signature (MM)	305.94	7.4	–9.2	7.6	207	0.66	0.79	0.12	74.8	2314	0.0004	BD	0.0020
KA2051A01-9	MM-310.3	Marine signature (MM)	310.25	7.5	–7.5	6.2	205	0.72	0.79	0.10	98.5	2792	0.0004	BD	0.0007
KA2050A-3	MM-318.9	Marine signature (MM)	318.78	7.5	–8.0	6.2	210	0.36	0.45	0.11	84.1	2509	0.0006	0.0003	0.0006
KA2051A01-5	MM-349.1	Marine signature (MM)	349.10	7.6	–9.0	7	190	0.22	0.22	0.12	68.3	2062	0.0003	BD	0.0011
KA2511A-5	MM-349.5	Marine signature (MM)	394.51	7.5	–7.9	4.9	156	0.47	0.32	0.16	103	3152	0.0005	0.0002	BD
KA3105A-4	MM-415.2	Marine signature (MM)	415.16	7.6	–8.8	5.5	149	0.31	0.40	0.11	83.2	2532	0.0004	BD	BD
KA3105A-3	MM-415.6	Marine signature (MM)	415.59	7.6	–8.6	5.4	147	1.04	0.28	0.15	82.5	2476	0.0003	BD	0.0005
HD0025A-1	MM-415.8	Marine signature (MM)	415.76	7.4	–8.7	4.6	143	0.72	1.50	0.08	131	3874	0.0003	0.0027	0.0015
KA3110A-1	MM-415.9	Marine signature (MM)	415.91	7.4	–7.9	5	148	0.26	1.22	0.10	121	3503	0.0004	0.0006	0.0033
KA2050A-1	MM-422.8	Marine signature (MM)	422.81	7.6	–8.6	6.1	191	0.59	0.59	0.07	87.8	2865	0.0003	BD	0.0005
KA3600F-2	MM-446.8	Marine signature (MM)	446.78	7.5	–8.6	3.6	99	0.18	0.29	0.06	108	4054	0.0005	0.0004	0.0005
KA3600F-1	MM-446.9	Marine signature (MM)	446.94	7.5	–8.8	3.4	73	0.09	0.17	0.03	108	4103	0.0002	0.0011	BD
KA3385A-1R	TM-448.4	Thoroughly mixed (TM)	448.35	7.5	–10.8	1.3	22	0.06	0.19	BD^d^	141	7489	0.0002	0.0010	0.0007
SA1730A-1	OS-237.0	Old saline (OS)	236.98	7.7	–12.2	1.1	13	0.04	0.18	BD	223	13,960	0.0002	0.0003	0.0007
KA1755A-3	OS-279.9	Old saline (OS)	279.89	7.8	–12.4	1	9	0.03	0.17	BD	218	13,350	0.0003	0.0007	0.0579
SA2600A-1	OS-345.0	Old saline (OS)	345.02	7.5	–12.2	1.4	19	0.05	0.19	BD	205	12,020	0.0004	BD	0.0010
KA2862A-1	OS-380.6	Old saline (OS)	380.63	7.8	–11.3	1.4	9	0.03	0.11	BD	207	16,220	0.0002	0.0003	0.0014

*Concentrations are shown in mg/L, except for depth, pH, and δ^*18*^O. Data shown for ${\mathrm{NO}}_{2}^{–}$, ${\mathrm{NO}}_{3}^{–}$, and ${\mathrm{PO}}_{4}^{3–}$ are average values from all measurements over the years, as values were below the detection limit in most of the measurements. ^*a*^Meters below sea level; ^*b*^pH units; ^*c*^‰; ^*d*^Below detection limit (S^*2*-^: 0.02 mg/L, ${\mathrm{NO}}_{2}^{–}$: 0.0002 mg/L, ${\mathrm{NO}}_{3}^{–}$: 0.0003 mg/L, and ${\mathrm{PO}}_{4}^{3–}$: 0.0005 mg/L).*

### Geochemical Analyses

The sampled boreholes were included in SKB’s geochemical monitoring of groundwaters at the Äspö HRL site, performed as described in Almén et al. (1986). In most cases, the monitoring started in the 1990s and has been carried out with variable frequency both within and between the boreholes. The rigorously quality controlled chemical and isotopic variables data were retrieved from SKB’s Sicada database (Data delivery SKBdata_17_061). These variables included (references are to analytical techniques and precision): Cl^-^ and δ^18^O that represent ^18^O/^16^O relative to Standard Mean Ocean Water (Mathurin et al., 2012); Ca, Mg, and ${\mathrm{NH}}_{4}^{+}$ (Mathurin et al., 2014b); Fe^2+^, DOC, and S^-^ (Alakangas et al., 2014); ${\mathrm{NO}}_{3}^{–}$ and ${\mathrm{NO}}_{2}^{–}$ (Rönnback and Åström, 2007); and Mn, ${\mathrm{SO}}_{4}^{2–}$, ${\mathrm{HCO}}_{3}^{–}$, and ${\mathrm{PO}}_{4}^{3–}$ (McMahon and Parnell, 2014). The extracted data are from three dates: (i) May 2016, which was as close as possible to the microbial sampling; (ii) May 2013, in order to assess water-quality stability in the short term; and (iii) the first sampling event in each borehole (in most cases in the 1990s) in order to assess long-term water-quality stability. One-way ANOVA *post hoc* test was used to look for significant differences (*p* < 0.05) in the chemical parameters between the three different water types. For this comparison, values from the last measurement were used except for nitrate, nitrite, and phosphate where an average of all measurements collected through the years was used as most measurements were close to or below the detection limit.

### Cell Numbers, DNA Content, and Size Distribution

To characterize the cell density, DNA content per cell, and cell size distribution, groundwaters were collected at the same time as for microbiological sampling. Triplicates of 1 mL of each of the 21 borehole section groundwaters were collected, fixed with 1% paraformaldehyde for 15 min at 4°C, then 5 mM EDTA and 2 g/L NaCl added, and the samples flash-frozen in liquid nitrogen. Triplicate samples were analyzed by flow cytometry after staining cells with the fluorescent dye Syto 13 (Molecular Probes, Invitrogen, Carlsbad, CA, United States) according to Giorgio et al. (1996). A flow cytometer equipped with a 488-nm blue solid-state laser (Cyflow Space, Partec, Görlitz, Germany) was used to determine cell counts, DNA content per cell, and size distribution. A volume of 50 μL was counted and green fluorescence was used as a trigger during the measurement. The flow cytometry counts were analyzed using Flowing Software version 2.5 (Perttu Terho, Center for Biotechnology, Turku, Finland). Median forward scatter was used as a proxy for average bacterial cell size and DNA content in individual cell samples (Bertilsson et al., 2003). This measurement does not provide information on absolute size without additional calibration, but allows the detection of relative differences in the cellular properties between the different samples. Coefficient of variation was calculated as the standard deviation of the replicates divided by the average. One-way ANOVA test was used to identify significant differences in cell numbers, DNA content per cell, and size distribution (p < 0.05) between the water types. Pearson bivariate correlation test was used to identify the correlation between cell numbers and DOC.

### Microbiological Sampling, DNA Extraction, Sequencing, and Bioinformatics

Groundwater sampling was carried out between February and May 2017. Before sampling, five section volumes of groundwater were discarded to avoid contamination from the stagnant water in the borehole pipe. Then, a high pressure, aluminum filter holder (Millipore) with a downstream valve and pressure gauge was directly connected to the borehole. Cell capture (in biological triplicates) was on a single sterile 0.1 μm pore size polyvinylidene fluoride (PVDF), hydrophilic, 25 or 47 mm Durapore Membrane filter (Merck Millipore) per replicate. The filters were aseptically removed from the holders, placed in cryotubes, and immediately frozen in liquid nitrogen for transport to the laboratory where they were stored at -80°C until further processing. The filtered volumes of groundwater per triplicate are shown in Supplementary Table S1. DNA extraction was performed using the MO BIO PowerWater DNA isolation kit following the manufacturer’s instructions except that the final DNA was re-suspended in 50 μL of eluent. The extracted DNA was analyzed by gel electrophoresis and a Qubit 2.0 Fluorometer (Life Technologies) and stored at -20°C until further processing. Then, the V3–V4 region of the 16S rRNA gene was amplified by a two-step PCR using bacterial primers 341F and 805R (Herlemann et al., 2011) and following the PCR protocol described in Hugerth et al. (2014) and followed by high-throughout sequencing at the Science for Life Laboratory, Sweden ^1^ on the Illumina MiSeq platform (2 × 300 bp pair-end reads). Finally, bioinformatic analyses were carried out as previously described (Lopez-Fernandez et al., 2018a). The number of pair-end reads received from the sequencing facility, merged, and quality trimmed reads, and amount of Operational Taxonomic Units (OTUs) clustered can be found in Supplementary Table S1. Final count values were normalized by relative abundance (i.e., % of total sample size) since it has been found to be more accurate than data rarefaction (McMurdie and Holmes, 2013). After normalization of counts, a one-way ANOVA *post hoc* test was used to (1) test the reproducibility of the biological triplicates per borehole section based on the 16S rRNA relative abundance of phyla and Proteobacteria classes (excluding former Epsilonproteobacteria; *p* < 0.05) in the three water types; (2) look for significant differences in 16S rRNA relative phyla and Proteobacteria classes abundance (*p* < 0.05) between the different water types; and (3) find significant differences for 16S rRNA gene relative classified OTUs abundance (*p* < 0.05) in the three different water types. Alpha diversity (Shannon H index) and Beta diversity (Bray–Curtis dissimilarity) were calculated based on rarefied data to the lowest sample size (9888 counts) and bootstrapped 100 times. Then, a one-way ANOVA test was used to look for significant differences in alpha diversity (*p* < 0.05) between the 21 different borehole waters. Finally, canonical correspondence analysis (CCA) was performed in PAST 3.17 (Hammer et al., 2001) to visualize the influence of the chemical parameters within the groundwaters and the differences in relative phyla abundance between the different groundwaters.

## Results and Discussion

### Geochemical Parameters of the Investigated Groundwaters

**FIGURE 2:**
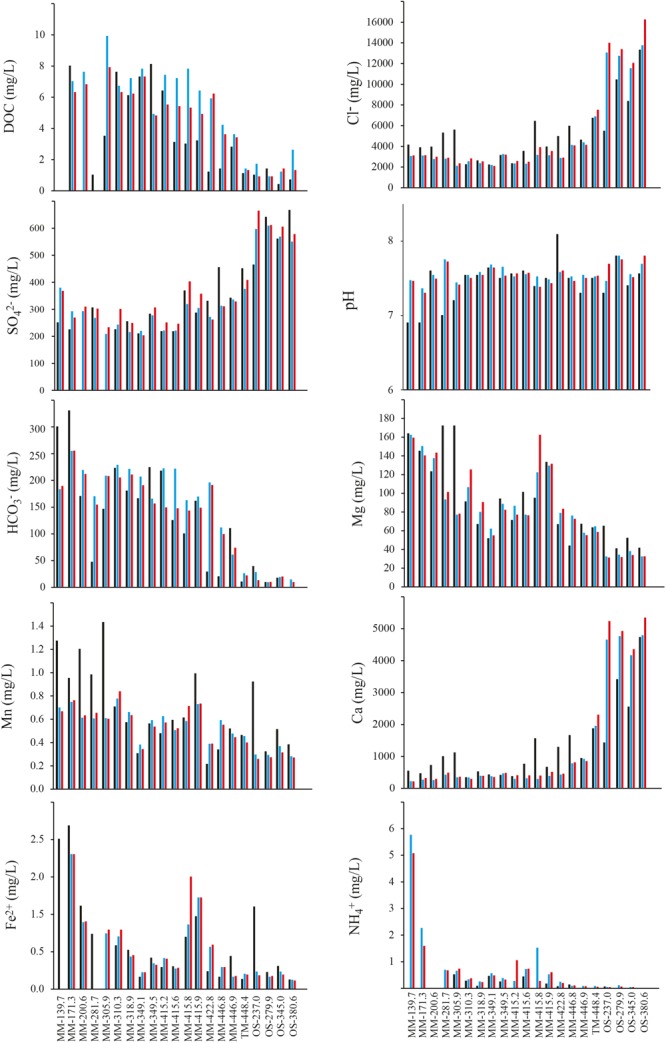
(Geo)chemical data for the 21 borehole sections. The black bar shows the first sampling occasion in the section (typically early/mid 1990s), the blue bar gives the value measured in approximately May 2013, and the red bar was measured in May 2016.

A time series of chemical parameters showing the stability of the sampled waters is shown in Figure 2 and Supplementary Table S2, while a summary of the geochemical parameters of the different groundwaters is given in Table 1. For 16 groundwaters, the recent (i.e., from 2013 to 2016) chloride concentrations and δ^18^O values were stable and ranged in total from 2000 to 4500 mg/L and from -7 to -10‰ vs. SMOW, respectively. These values are similar to the corresponding values in the Baltic Sea (3380 mg/L and -6‰ vs. SMOW) reported by Mathurin et al. (2012). Consequently, these groundwaters have a marine signature and are thus most likely composed of Baltic Sea water mixed with minor proportions of meteoric water and/or older more saline water residing in the bedrock fractures (Laaksoharju et al., 2008b). This interpretation was consistent with that the sampled bedrock volume was located under the archipelago on the western shores of the Baltic Sea and is characterized by dominantly sub-vertical fractures that allow for recharge of the overlying marine water (Mathurin et al., 2014b). However, the majority of these 16 groundwaters had higher chloride concentrations during the first sampling event (Figure 2 and Supplementary Table S2). This feature is ascribed to effects caused by the construction of the Äspö HRL tunnel in the 1990s. During the pristine conditions before tunnel construction, many of the fractures in contact with the overlying Baltic Sea were dominated by sea water intrusion during earlier stages of the Baltic Sea when the chloride concentrations were up to 6500 mg/L (Mathurin et al., 2012). After the tunnel construction, water flowing into and pumped away from the tunnel allowed for more efficient marine-water recharge leading to replacement of the ancient more saline waters with current Baltic Sea water (Mathurin et al., 2012). In consequence, at the time of sampling for microbiological analyses much of these groundwaters had recently intruded into the fracture network (within years or decades) and to some extent mixed with the equally young fresh (meteoric) water or older more saline waters previously occupying the fractures and therefore were named modern marine (MM) waters. Four of the groundwaters had considerably higher chloride concentrations and lower δ^18^O values (Figure 2 and Table 1). These groundwaters represent saline water that typically resides deep in fractured bedrock and are of up to millions of years old (Laaksoharju et al., 2008b), so they were termed OS waters. In contrast to the MM waters, these OS groundwaters increased in salinity since the first sampling (Figure 2). This has been explained by up-flow of very saline deep-lying water as a result of the abundant water discharge via the Äspö HRL tunnel (Laaksoharju et al., 1999). The remaining groundwater had chloride concentrations and δ^18^O values in-between the groundwaters with marine signature and the saline groundwaters (Figure 2 and Table 1). This groundwater is thus classified as thoroughly mixed (TM) and is composed of unknown proportions of two or more water types such that the age of this groundwater cannot be assessed.

The other determined chemical variables are non-conservative and can thus participate in various biogeochemical reactions within the fracture network, including reactions between the water and the fracture walls typically covered with secondary minerals such as calcite, clay minerals, and pyrite (Drake et al., 2013). Despite this, the concentrations of many of them were to a large extent controlled by water source and subsequent water-mixing processes within the fracture network (Gomez et al., 2014). Sulfate and calcium closely matched the chloride concentration and are much higher in the OS groundwaters (Figure 2). In contrast, magnesium, bicarbonate, DOC, and ammonium are higher in marine water than deep saline waters and consequently were higher in the MM groundwaters (Table 1 and Supplementary Table S2). A similar trend was seen for sulfide, which was below the detection limit (0.02 mg/L) in the OS groundwaters but was present in the MM waters at up to nearly 0.2 mg/L. Manganese and ferrous iron varied considerably among the boreholes but were not consistently different between the OS and MM groups (Table 1 and Supplementary Table S2). Finally, nitrite, nitrate, and phosphate occurred in very low concentrations at below (or very close to) the detection limit (Table 1) in the three different water types.

Almost all the tested chemical parameters were significantly different in the three groundwater types (MM, TM, and OS), as shown by the one-way ANOVA test (Supplementary Table S3). For example, Fe^2+^, DOC, S^2-^, and ${\mathrm{NO}}_{2}^{–}$ were significantly higher in the MM waters compared to the TM and OS boreholes (Supplementary Table S4). In addition, the concentrations of Cl^-^ and ${\mathrm{SO}}_{4}^{2–}$ were significantly higher in the OS waters compared to those in the MM and TM water types while in turn the values in the TM water were significantly higher compared to the MM waters. Moreover, the ${\mathrm{NO}}_{3}^{–}$ values were significantly higher only in the TM water. Finally, ${\mathrm{HCO}}_{3}^{–}$ was significantly different in all waters with highest values in the MM waters (Supplementary Table S4).

### Cell Numbers, DNA Content, and Size Distributions

**FIGURE 3:**
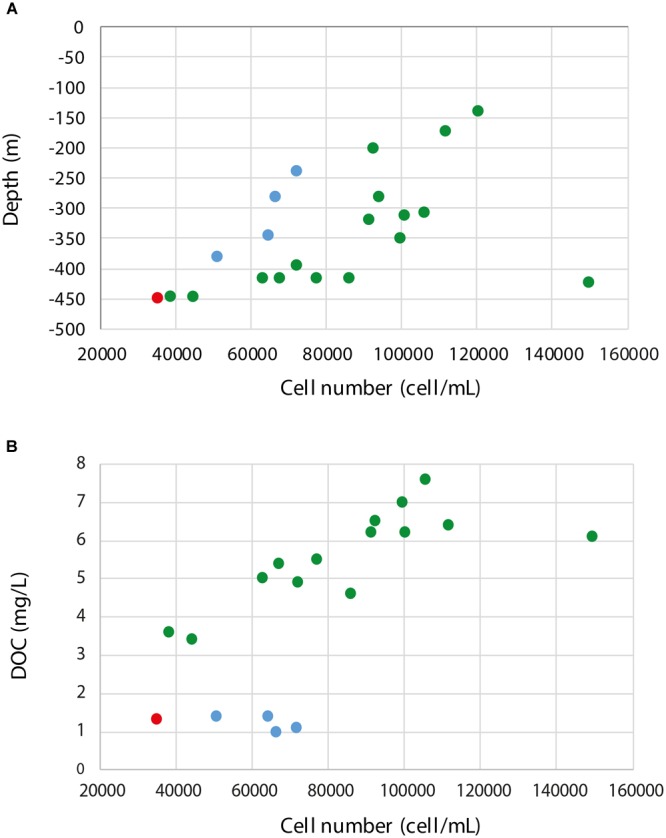
Pearson correlation plots **(A)** between the cell numbers and depth and **(B)** cell numbers and DOC (boreholes MM-139.7 and MM-281.7 have been omitted as no data for DOC were available) of the sampled borehole sections. MM waters are colored in green, TM in red, and OS in blue.

Cell counts, size distribution, and DNA content per cell are shown in Table 2 and Supplementary Figure S1. However, although cell size and cellular DNA content values were measured, they were close to the detection limit of the flow cytometer and both parameters feature high coefficient of variation. Significant differences were found in cell counts and size within the 21 borehole sections, but not in average cellular DNA content (*p* = 0.000, 0.004, and 0.100, respectively; *n* = 21). The flow cytometry analysis showed very low cell numbers compared to marine subsurface sediments with a range of 10^4^–10^8^ cells cm^-3^ (Morono et al., 2013), continental subsurface counts nearing 10^7^ cells cm^-3^ at depths down to 500 m below the surface (McMahon and Parnell, 2014), and sub-seafloor sediments (Kallmeyer et al., 2012). The data also revealed a significant positive correlation between decreasing cell numbers and depth (Pearson correlation = 0.50, *p* = 0.021, *n* = 21; Figure 3A). This has also been observed in a meta-study of over 100 continental deep biosphere cell density measurements (McMahon and Parnell, 2014). Interestingly, one exception to the trend was groundwater MM-422.81 that had higher cell numbers as well as an increased DOC content (Table 2 and Supplementary Figure S1) that again supports that the microbial cell numbers were linked to depth and DOC content.

**Table 2:** Average cell counts, DNA content per cell, and size distribution of the 21 sampled groundwaters measured by flow cytometry.

Borehole	Designation	Cell number (cell/mL ± SD^a^)	DNA content (plus CV)^b^	Cell size (plus CV)^c^
SA1009B-1	MM-139.7	120,333 ± 35,824	1.45 (322)	25.46 (250)
SA1229A-1	MM-171.3	111,583 ± 1130	0.42 (205)	13.11 (278)
SA1420A-1	MM-200.6	92,483 ± 7986	0.41 (299	10.06 (309)
KA2074A-1	MM-281.7	93,783 ± 3000	0.47 (194)	14.64 (268)
SA2273A-1	MM-305.9	105,750 ± 2555	0.43 (254)	9.88 (331)
KA2051A01-9	MM-310.3	100,450 ± 6848	0.47 (214)	11.69 (321)
KA2050A-3	MM-318.9	91,367 ± 5937	0.47 (263)	12.22 (300)
KA2051A01-5	MM-349.1	99,600 ± 2519	0.48 (217)	11.19 (296)
KA2511A-5	MM-349.5	71,967 ± 4763	0.50 (268)	14.04 (279)
KA3105A-4	MM-415.2	77,217 ± 1701	0.46 (240)	12.97 (285)
KA3105A-3	MM-415.6	67,283 ± 2032	0.52 (387)	12.98 (279)
HD0025A-1	MM-415.8	86,017 ± 4193	0.39 (307)	12.15 (254)
KA3110A-1	MM-415.9	62,983 ± 7279	0.42 (276)	12.76 (269)
KA2050A-1	MM-422.8	149,617 ± 4925	0.65 (231)	18.24 (256)
KA3600F-2	MM-446.8	38,283 ± 6876	0.50 (207)	13.32 (274)
KA3600F-1	MM-446.9	44,500 ± 1815	0.46 (261)	12.70 (269)
KA3385A-1R	TM-448.4	35,100 ± 1751	0.37 (203)	20.59 (224)
SA1730A-1	OS-237.0	71,783 ± 9408	0.32 (173)	14.62 (225)
KA1755A-3	OS-279.9	66,267 ± 4000	0.37 (314)	17.25 (237)
SA2600A-1	OS-345.0	64,417 ± 7438	0.35 (186)	14.93 (241)
KA2862A-1	OS-380.6	50,917 ± 3658	0.40 (246)	19.23 (227)

*^*a*^Standard deviation. ^*b*^DNA content per cell as arbitrary units with the coefficient of variation (%) in parenthesis. ^*c*^Cell size as arbitrary units with the coefficient of variation (%) in parenthesis.*

Flow cytometry also revealed a positive correlation between increased cell numbers and DOC (Pearson correlation = 0.17, *p* = 0.487, *n* = 19; Figure 3B) that was much stronger for the MM (Pearson correlation = 0.20, *p* = 0.496, *n* = 14) compared to the OS (Pearson correlation = -0.03, *p* = 0.973, *n* = 4) fracture waters. This also supported that MM waters depend on organic carbon sources infiltrated from the surface while the OS waters were fed from below by carbon dioxide and hydrogen of geological origin (Fry et al., 2008; Pedersen, 2013; Wu et al., 2015; Lopez-Fernandez et al., 2018b).

A small cell size of <0.2 μm and a streamlined genome are suggested to be adaptation strategies to the oligotrophic conditions in the deep biosphere (Giovannoni et al., 2014; Wu et al., 2015). Interestingly, the shallowest water included in this survey (groundwater MM-139.7) showed the highest values for DNA content per cell and cell size (Figure 3), which might be related to the proximity of this groundwater to the surface and the influence of the brackish water penetrating from the Baltic Sea. Assuming the limitations of the DNA content measurements and the possibility of multiple genome copies per cell, the varying relative proportions of populations with streamlined genomes between the water types may also explain the lack of a correlation between cell numbers and average cellular DNA content (Supplementary Figure S1).

### Microbial Diversity Analyses

**FIGURE 4:**
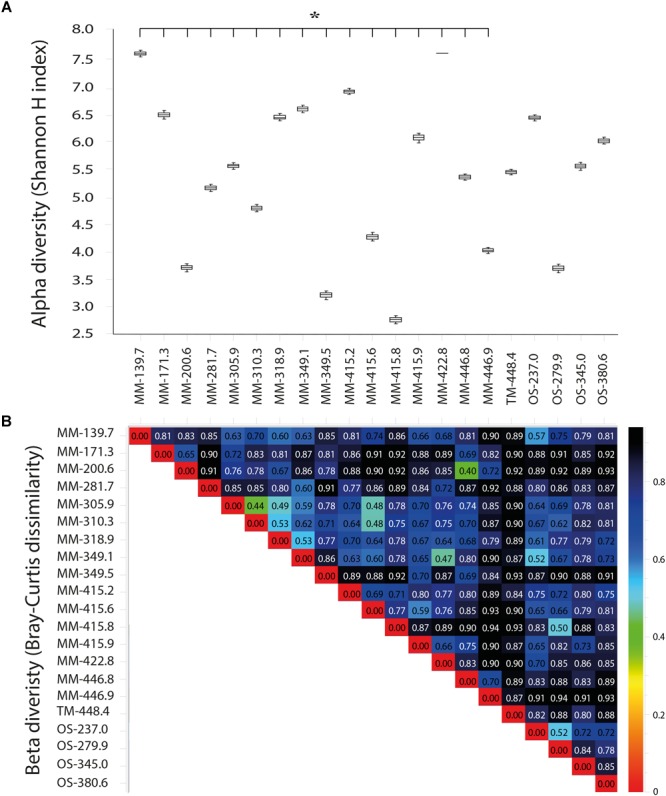
Shannon H alpha **(A)** and Bray–Curtis **(B)** diversities based on OTUs from the 21 sampled boreholes sections. The statistically significant Shannon H alpha diversity (*p* < 0.05) is marked with a star.

Details of the water volume filtered, amount of DNA extracted, and number of 16S rRNA gene amplicons obtained from the sequencing of the 21 boreholes are provided in Supplementary Table S1. The reproducibility of the biological triplicate samples per borehole section was confirmed as differences between each of the triplicates were insignificant (one-way ANOVA test, *p* > 0.05, *n* = 3 per borehole section; Supplementary Table S5). No statistically significant differences were found in the alpha diversity (Shannon H index) when comparing all the 21 borehole waters to each other (one-way ANOVA test, *p* > 0.05, *n* = 3 per borehole section; Figure 4A). In contrast, significant increases in alpha diversity were found when comparing the 16 MM waters to the single TM and four OS boreholes (one-way ANOVA *post hoc* test, *p* = 0.00, *n* = 48 for MM, *n* = 3 for TM and *n* = 12 for OS) supporting a greater diversity in the microbial communities closer to the surface and with more DOC content. However, no significant differences were found when comparing within the four OS waters (Figure 4A). Beta diversity analysis (Bray–Curtis dissimilarity) revealed that the three water types contained different microbial populations, with the highest dissimilarity in the TM water compared to the others (Figure 4B). This was likely due to the special chemical composition of this groundwater type composed by a mix of waters.

### Microbial Community Composition in the Water Types

**FIGURE 5:**
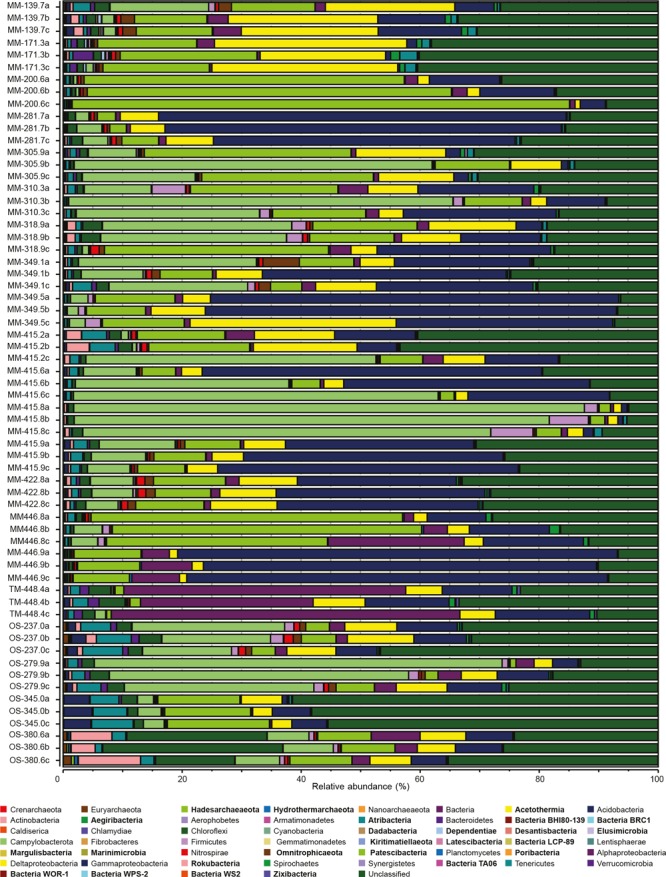
Relative abundances of triplicate 16S rRNA gene OTUs sequences from the 21 borehole sections. Microbial community is shown at phylum level (except for Proteobacteria, which have been divided into classes). Candidate phyla are labeled in bold type.

The dominant identified microbial taxa in all three groundwater types (Figure 5 and Supplementary Table S6) were Gammaproteobacteria (23% of the total relative abundance), unclassified sequences (22%), the newly proposed Campylobacterota (18%, formerly Epsilonproteobacteria) (Waite et al., 2017, 2018), Patescibacteria (14%), Delta- (7%), and Alphaproteobacteria (6%). At the OTU level, 16S rRNA gene sequences most similar to *Sulfurimonas* totaled 35.7 and 20.2% in the MM and OS waters, respectively; sequences most similar to the *Thiobacillus* genus constituted 21.2% of the MM waters; and sequences aligning with the genus *Filomicrobium* were dominant in the TM water with 34.6%. The deeper OS waters also showed a significantly higher relative abundance of Archaea including Euryarchaeota, Nanoarchaeota, Hadesarchaeaota, and the recently identified Hydrothermarchaeota (one-way ANOVA *post hoc* test, *p* < 0.05, *n* = 48 for MM, *n* = 3 for TM, and *n* = 12 for OS). The increased relative proportion of archaea was in agreement with other studies that reveal a dominance of Archaea in deep subsurface sediments (Biddle et al., 2006; Teske and Sørensen, 2008).

Not surprisingly for the extremely oligotrophic and the largely unexplored deep biosphere, many OTUs were affiliated to candidate phyla and unclassified microorganisms. Almost all candidate phyla that were significantly different between the three different water types showed a higher relative abundance in the OS waters (Figure 5 and Supplementary Table S4). In detail, 11 of the 21 groundwaters had >20% of unclassified OTUs, with the highest representation in borehole OS-345.0 with an average between the triplicate samples of >58% unclassified sequences. In addition, a total of 36 OTUs (10 in the MM, 18 in the OS, and 8 in the TM waters, Supplementary Table S6) had significantly increased relative abundances (one-way ANOVA *post hoc* test, *p* < 0.05, *n* = 48 for MM, *n* = 3 for TM, and *n* = 12 for OS) that aligned within the Patescibacteria superphylum. The newly described Patescibacteria may include more than 35 candidate phyla with reduced metabolic capacities (Hug et al., 2016; Parks et al., 2017). Despite that the Tree of Life has recently been expanded with many candidate phyla identified from shallow subsurface waters (Castelle et al., 2015;

Anantharaman et al., 2016, 2018; Hug et al., 2016), the continental deep biosphere contains a large microbial diversity waiting to be characterized and this proportion increases with depth and temporal separation from the surface. In addition, a more thorough study is required to elucidate if those populations placed within known taxa (e.g., the high relative proportion of Proteobacteria, excluding in this study the newly proposed phylum Campylobacterota, formerly Epsilonproteobacteria) are comprised of novel, deep subsurface adapted species.

### Water Chemistry Shapes the Microbial Community Metabolic Capacities

**FIGURE 6:**
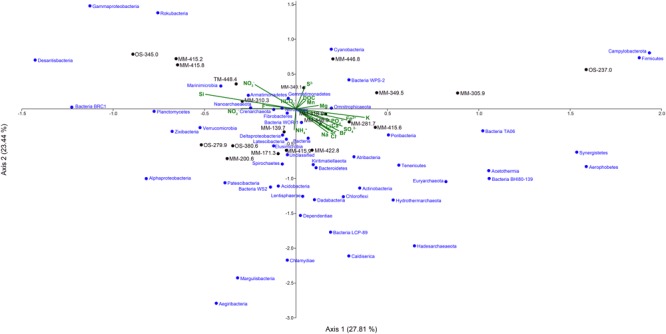
Canonical correspondence analysis of the 16S rRNA gene OTUs at phylum level (except for Proteobacteria, which have been divided into classes) combined with the geochemical data from the 21 borehole sections.

The CCA based on the chemical parameters and 16S rRNA gene relative abundances supported that the water chemistries influenced the microbial diversity and that there were differences between the MM, TM, and OS waters (Figure 6 and Supplementary Table S4). The CCA showed that the higher DOC containing MM waters were separated into two groups based on the influence of nitrate/nitrite or ferrous iron (Figure 6), suggesting that nitrogen and iron cycling were major processes in the MM waters. Nitrite and ammonia were significantly higher in the MM waters compared to the OS and TM, while nitrate was statistically increased only in the TM water (one-way ANOVA *post hoc* test, *p* < 0.05, *n* = 48 for MM, *n* = 3 for TM, and *n* = 12 for OS; Supplementary Table S4). Many nitrogen-cycling taxa have been previously identified in Fennoscandian fracture waters (Kutvonen et al., 2015; Sohlberg et al., 2015; Hubalek et al., 2016; Purkamo et al., 2016). In addition, the genetic potential to fix nitrogen by a microbial community in a granitic subsurface mine has been described and that fixed nitrogen from a biological origin may support subsurface biomass (Swanner and Templeton, 2011). In addition, previous reconstruction of metagenome assembled genomes from the Äspö HRL continental deep subsurface showed a higher than anticipated prevalence of nitrate reducing populations (Wu et al., 2015). Those microorganisms present in this study included *Sulfurimonas* (Han and Perner, 2015) that despite constituting 35.7% of the total MM population did not have any OTUs with a statistically higher relative proportion compared to the OS and TM waters. However, *Sulfurimonas* are commonly isolated from sulfidic habitats and are also able to use reduced sulfur compounds as electron donor (Han and Perner, 2015). Therefore, the identified OTUs aligning with *Sulfurimonas* in this study might be involved in both the sulfur and nitrogen cycles. In contrast to the nitrate reducers in the MM waters, 4 Nitrospirae OTUs, 10 *Pseudomonas* OTUs, and 6 Hadesarchaeaeota OTUs were significantly higher (one-way ANOVA *post hoc* test, *p* < 0.05, *n* = 48 for MM, *n* = 3 for TM, and *n* = 12 for OS; Supplementary Table S6) in the OS water compared to the MM waters. Finally, the TM water contained seven OTUs aligning with the *Thiobacillus* genus that had significantly higher relative proportions compared to one or both of the MM and OS waters (one-way ANOVA *post hoc* test, *p* < 0.05, *n* = 48 for MM, *n* = 3 for TM, and *n* = 12 for OS; Supplementary Table S6). A potential product of nitrate reduction is ammonium that can then be assimilated as a nitrogen source within the cell, such as by the newly proposed phylum Campylobacterota (formerly Epsilonproteobacteria). Although there were no statistically significant differences between the three studied groundwater types at the phylum level, this new taxa showed a high relative abundance in the MM waters, especially in the MM-415.8 groundwater where the average relative abundance was greater than 78% (Figure 5). In addition, the TM water had a significantly increased ammonium concentration compared to the other waters (one-way ANOVA *post hoc* test, *p* < 0.05, *n* = 48 for MM, *n* = 3 for TM, and *n* = 12 for OS; Supplementary Table S4). The phyla with significantly higher relative abundances in the TM-448.4 included candidate (Ca.) phylum Zixibacteria, formerly RBG-1 (one-way ANOVA *post hoc* test, *p* < 0.05, *n* = 48 for MM, *n* = 3 for TM, and *n* = 12 for OS; Supplementary Table S4) that has been identified as the dominant microorganisms in aquifer sediments and has the ability to assimilate ammonium (Castelle et al., 2013). Previously, the deep continental biosphere was suggested to be mainly supported by sulfate reduction (Nielsen et al., 2006; Hallbeck and Pedersen, 2008; Katsuyama et al., 2013). However, although nitrate, nitrite, and ammonium concentrations were very low in this oligotrophic subsurface environment and regardless of their ultimate source (Swanner and Templeton, 2011), this study also supports that nitrogen cycling may be important for the microorganisms inhabiting the deep biosphere (Kutvonen et al., 2015).

Ferrous iron was significantly higher in the MM compared to the other two water types (one-way ANOVA *post hoc* test, *p* = 0.001, *n* = 48 for MM, *n* = 3 for TM, and *n* = 12 for OS; Supplementary Table S4), suggesting that anaerobic ferric iron reducing microorganisms contributed to the increased level of ferrous iron in this water type. Microbial ferric iron reduction is phylogenetically scattered throughout the bacterial and archaeal domains, but mainly mediated by the Geobacteraceae family in subsurface environments (Lovley et al., 2004). The presence of ferrous-oxidizing and ferric-reducing microorganisms was identified using flow cells at the Äspö HRL (Ionescu et al., 2015). In this study, taxa that potentially carry out ferric reduction included two OTUs (one of them identified in all three water types) affiliated to *Shewanella* and with higher but not significantly different relative abundance in the MM waters (Supplementary Table S6); and one OTU from the order Desulfuromonadales (containing the *Geobacter* genus) identified as significantly higher in the OS compared to MM waters (one-way ANOVA *post hoc* test, *p* = 0.012, *n* = 48 for MM, *n* = 3 for TM, and *n* = 12 for OS; Supplementary Table S6). Ferrous iron-oxidizing taxa included the *Ferritrophicum* genus (Weiss et al., 2007) and Gallionellaceae family (Hallbeck and Pedersen, 2014) with 14 and six OTUs comprising 10.1 and 1.7% of the MM relative populations, respectively. Although the MM groundwaters contained a higher relative proportion of *Ferritrophicum*, the individual OTUs were not significantly increased compared to the OS and TM waters (Supplementary Table S6). Although the characterized species from these genera are microaerobic, they were identified in the studied anaerobic groundwaters and have also been described to be able to conserve energy under anaerobic conditions (He et al., 2016).

Sulfate was significantly higher in the OS compared to the other two water types while sulfide was significantly higher in the MM waters (one-way ANOVA *post hoc* test, *p* < 0.05, *n* = 48 for MM, *n* = 3 for TM, and *n* = 12 for OS; Supplementary Table S4). The higher concentration of sulfate and sulfide in the OS and MM waters, respectively, likely reflects the relative availability of electron donors in the two groundwater types. Deltaproteobacteria have been previously described as sulfate reducers in deep biosphere environments (e.g., Orsi et al., 2013). Via their ability to also grow by disproportionation of inorganic sulfur compounds (i.e., using them as both electron donor and acceptor) they generate hydrogen sulfide and sulfate (Finster, 2008), and this suggests sulfur cycling was occurring in this extremely oligotrophic environment. Deltaproteobacteria were highly represented in the three different water types and had the highest average relative abundances in MM-139.7 and MM-173.3 with 23.3 and 21.1%, respectively (Figure 5). Only a few OTUs assigned to the Deltaproteobacteria had significantly increased relative abundances in the MM waters (Supplementary Table S6) and these included 16S rRNA gene sequences that were assigned to the Desulfobacteraceae family and *Desulfatiglans* genus (one-way ANOVA *post hoc* test, *p* < 0.05, *n* = 48 for MM, *n* = 3 for TM, and *n* = 12 for OS). Members of the *Desulfatiglans* genus (Suzuki et al., 2014) and most of the Desulfobacteraceae (Kuever, 2014) are heterotrophs that match the reliance of the MM waters on organic carbon infiltration. That all four OS waters clustered with low DOC and high sulfate concentration (Figure 6) support the previous paradigm of a hydrogen-fueled, sulfate reducing deep biosphere community (Hallbeck and Pedersen, 2008; Wu et al., 2017) that is fed by organic carbon of a microbial origin (Purkamo et al., 2015; Kieft et al., 2018). This is supported by three *Desulfocarbo* OTUs suggested to be able to grow autotrophically with hydrogen (Kuever, 2014), two *Desulfovibrio* OTUs that potentially use hydrogen as electron donor (Kushwaha et al., 2018), three *Desulfurivibrio* OTUs that may use hydrogen (among others) as electron donor coupled to thiosulfate/polysulfide as electron acceptor (Sorokin et al., 2008), four *Sulfurimonas* OTUs that are described for their versatile chemoautotrophic metabolism (Han and Perner, 2015) that were significantly higher in the OS waters (one-way ANOVA *post hoc* test, *p* < 0.05, *n* = 48 for MM, *n* = 3 for TM, and *n* = 12 for OS; Supplementary Table S6). Sulfur oxidizing bacteria and archaea are also present in subsurface habitats (Ghosh and Dam, 2009). Sulfide ion (predominantly H_2_S/HS^-^) was significantly higher in the MM waters (one-way ANOVA *post hoc* test, *p* = 0.05, *n* = 48 for MM, *n* = 3 for TM, and *n* = 12 for OS; Supplementary Table S4) where the relative abundance of Gammaproteobacteria was also significantly higher (one-way ANOVA *post hoc* test, *p* = 0.006, *n* = 48 for MM, *n* = 3 for TM, and *n* = 12 for OS; Figure 5 and Supplementary Table S4). Some Gammaproteobacteria have been highlighted for their role in sulfur oxidation in deep sea hydrothermal vents (Sievert et al., 2008). One highly represented genus from the Gammaproteobacteria was the sulfur compound-oxidizing *Thiobacillus* (Robertson and Kuenen, 2006) that totaled 21.2% of the MM relative population but only one OTU was significantly increased compared to the OS water (one-way ANOVA *post hoc* test, *p* = 0.007, *n* = 48 for MM, *n* = 3 for TM, and *n* = 12 for OS; Supplementary Table S6). Moreover, Ca. phylum Marinimicrobia (SAR406), recently described for its implication in the ocean sulfur cycling via the polysulfide reductase ABC complex that can use H_2_S as an auxiliary electron donor (Hawley et al., 2017), was significantly higher in the MM waters (one-way ANOVA *post hoc* test, *p* = 0.048, *n* = 48 for MM, *n* = 3 for TM, and *n* = 12 for OS; Figure 5 and Supplementary Table S4). This supports that cryptic sulfur cycling was occurring in the three water types and that microbial sulfur disproportionation might be an option to deal with the limitation of electron donor, which may also explain the lack of accumulation of reduced sulfur compounds.

Sources of methane in the deep crystalline bedrock can be both abiotic (e.g., Ischer–Tropsch-type methane production) or biotic via methanogenesis (Kietäväinen and Purkamo, 2015). Methanogenesis is solely carried out by the Archaea and these microbes have been identified in groundwaters from the Fennoscandian Shield (Pande et al., 2014; Wu et al., 2015). In this study, three OTUs that align within the family Methermicoccaceae (Oren, 2014b) and one OTU from the Methanobacteriaceae (Oren, 2014a) had higher relative abundances in the OS water compared to MM groundwaters (one-way ANOVA *post hoc* test, *p* < 0.05, *n* = 48 for MM, *n* = 3 for TM, and *n* = 12 for OS; Supplementary Table S6). In addition, methanogenic Archaea from the Methanomassiliicoccales (Soellinger et al., 2015) were present in all waters, but no statistically significant differences were observed between the three different groundwaters. Although methane measurements were not available and therefore, its cycling cannot be confirmed the presence of OTUs that aligned with methane oxidizers and reducers suggested it played a role in the Äspö HRL groundwaters. Anaerobic oxidation of methane can be linked to nitrate reduction by the Ca. genus *Methanoperedens* (Vaksmaa et al., 2017) that was present in all three groundwaters with a low relative abundance. A second method of anaerobic methane oxidation is via syntrophic associations between microorganisms degrading organic compounds and methanogens. This is the case for one OTU from the genus *Syntrophorhabdus* (Qiu et al., 2008), 15 OTUs from the family Anaerolineaceae (McIlroy et al., 2017), and three OTUs similar to the genus *Syntrophus* (Siddique et al., 2011) that were significantly increased in the MM compared to the other two groundwaters (one-way ANOVA *post hoc* test, *p* < 0.05, *n* = 48 for MM, *n* = 3 for TM, and *n* = 12 for OS; Supplementary Table S6). These syntrophic relationships of symbiotic taxa that grow alongside methanogens supported that the MM waters have a community with more varied metabolic strategies compared to the OS waters (Wu et al., 2015; Lopez-Fernandez et al., 2018b). In addition, methanogens were represented by one OTU aligning to *Methylobacterium* genus (Stein, 2018) that was significantly higher in the OS waters, and three OTUs from the genus *Methylomicrobium* (Akberdin et al., 2018) significantly higher in the TM water (one-way ANOVA *post hoc* test, *p* < 0.05, *n* = 48 for MM, *n* = 3 for TM, and *n* = 12 for OS; Supplementary Table S6). Finally, five OTUs were significantly higher in the OS waters compared to the MM and TM (one-way ANOVA *post hoc* test, *p* < 0.05, *n* = 48 for MM, *n* = 3 for TM, and *n* = 12 for OS; Supplementary Table S6) aligning to the Ca. phylum Atribacteria (OP9) (Carr et al., 2015), which can potentially produce fermentation products that may also support methanogens. That OTUs assigned to archaeal and bacterial taxa carrying out methanogenesis had significantly higher relative abundance in the OS groundwaters supports the extreme oligotrophic conditions in these waters fed by geogases coming from below (Wu et al., 2015; Lopez-Fernandez et al., 2018b).

## Conclusion

The 16S rRNA gene relative abundance of the 21 studied groundwaters showed dissimilar microbial community compositions, with significant differences between the three different water types. Specifically, the microbial diversity within the waters with MM signature was higher in the groundwaters with more DOC content. The TM water showed the highest dissimilarity compared to the other two water types, probably due to the more different chemical composition of this water type. Based on the chemical composition of the three classified water types, the identified microbial phyla might be involved in cycling of nitrogen, iron, and sulfur. Finally, the relative proportion of unclassified microorganisms and candidate phyla increased with depth. This indicates the importance and necessity of further studies to characterize the deep biosphere microbial populations and their influence in the global nutrients and energy cycles.

## Data Availability

16S rRNA gene sequences are available at NCBI database with the Bioproject accession number PRJNA434543.

## Author Contributions

ML-F, MD, and SB conceived the study. ML-F collected samples, carried out laboratory work, and analyzed biological data. MÅ analyzed geochemical data. ML-F drafted the manuscript and all authors gave final approval for publication.

## Conflict of Interest Statement

The authors declare that the research was conducted in the absence of any commercial or financial relationships that could be construed as a potential conflict of interest.

## Footnotes

1 www.scilifelab.se

## Supplementary Material

FIGURE S1 Average cell numbers including error bars **(A)**, DNA content per cell **(B)**, and size distribution **(C)** of triplicate measurements, including standard deviation and coefficient of variation, of the microbial community from the 21 borehole sections. DNA content and size distribution are shown in arbitrary units.

TABLE S1 Sampling and sequencing information of 21 borehole sections representing the borehole and sample name, water type, total volume of water sampled, DNA extracted, number of read pairs obtained from the sequencing facility, after merging and quality trimming, and the amount of OTUs.

TABLE S2 Detailed geochemical composition of the 21 groundwaters since the monitoring started in early/mid-1990s until the last measurement in May 2016 (November 2015 only for SA2074A-1).

TABLE S3 One-way ANOVA of the difference in the chemical values between the three water types (*p*-values are shown in the table). Average values (mg/L, except for pH in units) for the chemical data in each water type are included. Differences in values that are statistically significant (*p* < 0.05) are marked in color.

TABLE S4 One-way ANOVA *post hoc* test of the difference in chemical values and 16S rRNA relative abundance for individual phyla and proteobacterial classes between the three water types (*p*-values are shown in the table). Statistically significant differences (*p* < 0.05) are marked in color with the water type with higher values noted.

TABLE S5 One-way ANOVA *post hoc* test of the difference in 16S rRNA gene relative abundance for individual phyla and proteobacterial classes between the triplicate samples per borehole section (*p*-values are shown in the table).

TABLE S6 Average relative abundance of the OTUs (97% similarity) for the three water types plus one-way ANOVA *post hoc* test of the difference in 16S rRNA gene relative abundance for all comparisons between the water types (i.e., MM vs. OS, MM vs. TM, and TM vs. OS). Significant differences are colored for the water type where the OTU has a significantly increased relative abundance (MM, green; TM, red; and OS, blue).
