# Supplementary figures and images for: Depth and Dissolved Organic Carbon Shape Microbial Communities in Surface Influenced but Not Ancient Saline Terrestrial Aquifers
Source: Front Microbiol. 2018 Nov 27;9:2880. doi: 10.3389/fmicb.2018.02880 (PMC6277548; doi:10.3389/fmicb.2018.02880)

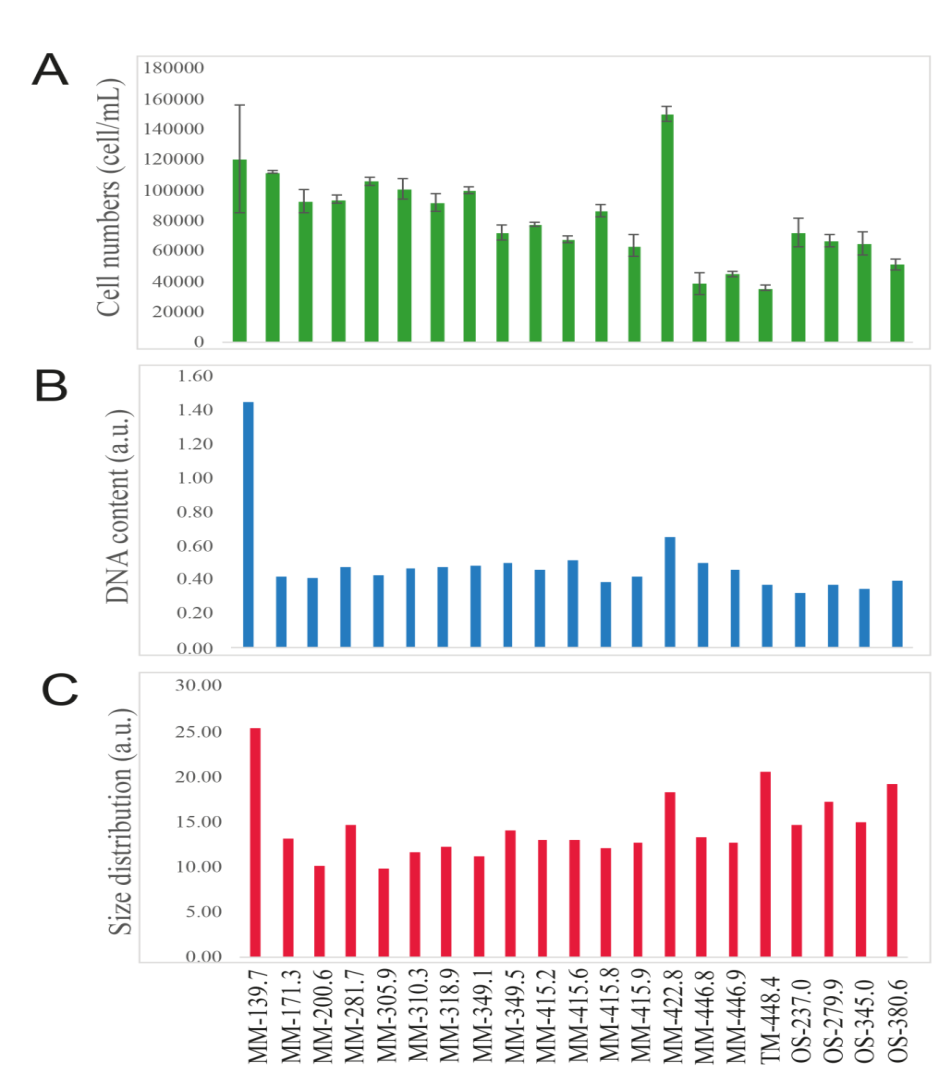

Supplement: FIGURE S1 — Average cell numbers including error bars (A), DNA content per cell (B), and size distribution (C) of triplicate measurements, including standard deviation and coefficient of variation, of the microbial community from the 21 borehole sections. DNA content and size distribution are shown in arbitrary units. [file Image_1.TIF]
