# Supplementary material for: Depth and Dissolved Organic Carbon Shape Microbial Communities in Surface Influenced but Not Ancient Saline Terrestrial Aquifers
Source: Front Microbiol. 2018 Nov 27;9:2880. doi: 10.3389/fmicb.2018.02880 (PMC6277548; doi:10.3389/fmicb.2018.02880)
Supplement: TABLE S3 — One-way ANOVA of the difference in the chemical values between the three water types (p-values are shown in the table). Average values (mg/L, except for pH in units) for the chemical data in each water type are included. Differences in values that are statistically significant (p < 0.05) are marked in color. [file Table_3.DOCX]

Supplementary Material

Depth and dissolved organic carbon shape microbial communities in surface influenced but not ancient saline terrestrial aquifers

Margarita Lopez-Fernandez^1,≠,*^, Mats Åström^2^, Stefan Bertilsson^3^, and Mark Dopson^1,*^

*** Correspondence:** [margarita.lopezfernandez@lnu.se](mailto:margarita.lopezfernandez@lnu.se) and [mark.dopson@lnu.se](mailto:mark.dopson@lnu.se)

**Supplemental Table 3.** One-Way ANOVA of the difference in the chemical values between the three water types (p-values are shown in the table). Average values (mg/L, except for pH in units) for the chemical data in each water type are included. Differences in values that are statistically significant (p < 0.05) are marked in color.

|  | MM | TM | OS | MM-TM-OS |
| --- | --- | --- | --- | --- |
| pH | 7.51 | 7.53 | 7.69 | 0.000 |
| Na | 2025.63 | 1710.00 | 2050.00 | 0.000 |
| K | 17.05 | 31.0 | 17.7 | 0.056 |
| Ca | 1506.2 | 501.0 | 1077.2 | 0.000 |
| Mg | 83.5 | 131.0 | 86.8 | 0.000 |
| HCO_3_^-^ | 144.5 | 148.1 | 84.02 | 0.000 |
| Cl^-^ | 5514.9 | 3503.0 | 4880.0 | 0.000 |
| SO_4_^2-^ | 358.5 | 355.7 | 361.1 | 0.000 |
| Br | 33.8 | 14.2 | 25.9 | 0.000 |
| F | 1.48 | 1.30 | 1.37 | 0.000 |
| Si | 5.86 | 5.55 | 5.90 | 0.000 |
| Fe(II) | 0.56 | 1.22 | 0.54 | 0.001 |
| Mn | 0.51 | 0.73 | 0.52 | 0.000 |
| Li | 1.07 | 0.28 | 0.78 | 0.000 |
| Sr | 26.47 | 8.23 | 18.54 | 0.000 |
| DOC | 4.81 | 4.90 | 3.40 | 0.000 |
| S^2-^ | 0.08 | 0.10 | 0.04 | 0.000 |
| NO_2_^-^ | 0.0004 | 0.0004 | 0.0003 | 0.000 |
| NO_3_^-^ | 0.0038 | 0.0005 | 0.0013 | 0.619 |
| NH_4_^+^ | 1.00 | 0.26 | 0.26 | 0.038 |
| PO_4_^3+^ | 0.013 | 0.003 | 0.001 | 0.386 |
