# Supplementary material for: Depth and Dissolved Organic Carbon Shape Microbial Communities in Surface Influenced but Not Ancient Saline Terrestrial Aquifers
Source: Front Microbiol. 2018 Nov 27;9:2880. doi: 10.3389/fmicb.2018.02880 (PMC6277548; doi:10.3389/fmicb.2018.02880)
Supplement: TABLE S4 — One-way ANOVA post hoc test of the difference in chemical values and 16S rRNA relative abundance for individual phyla and proteobacterial classes between the three water types (p-values are shown in the table). Statistically significant differences (p < 0.05) are marked in color with the water type with higher values noted. [file Table_4.DOCX]

Supplementary Material

Depth and dissolved organic carbon shape microbial communities in surface influenced but not ancient saline terrestrial aquifers

Margarita Lopez-Fernandez^1,≠,*^, Mats Åström^2^, Stefan Bertilsson^3^, and Mark Dopson^1,*^

*** Correspondence:** [margarita.lopezfernandez@lnu.se](mailto:margarita.lopezfernandez@lnu.se) and [mark.dopson@lnu.se](mailto:mark.dopson@lnu.se)

**Supplemental Table 4.** One-Way ANOVA posthoc test of the difference in chemical values and 16S rRNA relative abundance for individual phyla and proteobacterial classes between the three water types (p-values are shown in the table). Statistically significant differences (p < 0.05) are marked in color with the water type with higher values noted.

|  | **MM-OS** | **MM-TM** | **OS-TM** |
| --- | --- | --- | --- |
| pH | 0.000 OS | 0.680 | 0.037 OS |
| Na | 0.000 OS | 0.000 TM | 0.000 OS |
| K | 0.053 | 0.146 | 0.026 OS |
| Ca | 0.000 OS | 0.000 TM | 0.000 OS |
| Mg | 0.000 MM | 0.039 MM | 0.000 TM |
| HCO_3_^-^ | 0.000 MM | 0.000 MM | 0.004 TM |
| Cl^-^ | 0.000 OS | 0.000 TM | 0.000 OS |
| SO_4_^2-^ | 0.000 OS | 0.000 TM | 0.000 OS |
| Br | 0.000 OS | 0.000 TM | 0.000 OS |
| F | 0.000 OS | 0.574 | 0.001 OS |
| Si | 0.000 MM | 0.002 MM | 0.019 TM |
| Fe(II) | 0.001 MM | 0.095 | 0.178 |
| Mn | 0.000 MM | 0.013 MM | 0.000 TM |
| Li | 0.000 OS | 0.000 TM | 0.000 OS |
| Sr | 0.000 OS | 0.000 TM | 0.000 OS |
| DOC | 0.000 MM | 0.000 MM | 0.237 |
| S^2-^ | 0.000 MM | 0.001 MM | NA^a^ |
| NO_2_^-^ | 0.000 MM | 0.030 MM | 0.708 |
| NO_3_^-^ | 0.347 | 0.794 | 0.000 TM |
| NH_4_^+^ | 0.021 MM | 0.252 | 0.001 TM |
| PO_4_^3+^ | 0.245 | 0.532 | 0.358 |
| Archaea/Crenarchaeota | 0.783 | 0.368 | 0.369 |
| Archaea/Euryarchaeota | 0.000 OS | 0.916 | 0.139 |
| Archaea/Hadesarchaeaeota | 0.000 OS | 0.818 | 0.322 |
| Archaea/Hydrothermarchaeota | 0.000 OS | 0.841 | 0.25 |
| Archaea/Nanoarchaeaeota | 0.106 | 0.156 | 0.031 OS |
| Bacteria | 0.82 | 0.108 | 0.151 |
| Bacteria/Acetothermia | 0.188 | 0.49 | 0.199 |
| Bacteria/Acidobacteria | 0.000 OS | 0.005 TM | 0.365 |
| Bacteria/Actinobacteria | 0.001 OS | 0.681 | 0.359 |
| Bacteria/Aegiribacteria | 0.25 | 0.565 | NA |
| Bacteria/Aerophobetes | 0.004 OS | 0.611 | 0.31 |
| Bacteria/Armatimonadetes | 0.106 | 0.328 | 0.484 |
| Bacteria/Atribacteria | 0.000 OS | 0.191 | 0.084 |
| Bacteria/Bacteroidetes | 0.178 | 0.000 TM | 0.002 TM |
| Bacteria/BHI80-139 | 0.000 OS | 0.632 | 0.112 |
| Bacteria/BRC1 | 0.064 | 0.352 | NA |
| Bacteria/Caldiserica | 0.182 | 0.925 | 0.04 TM |
| Bacteria/Chlamydiae | 0.346 | 0.57 | 0.635 |
| Bacteria/Chloroflexi | 0.000 OS | 0.000 TM | 0.491 |
| Bacteria/Cyanobacteria | 0.09 | 0.617 | 0.109 |
| Bacteria/Dadabacteria | 0.547 | 0.717 | 0.635 |
| Bacteria/Dependentiae | 0.193 | 0.427 | 0.34 |
| Bacteria/Desantisbacteria | 0.25 | 0.565 | NA |
| Bacteria/Elusimicrobia | 0.048 OS | 0.000 TM | 0.022 TM |
| Bacteria/Epsilonbacteraeota | 0.659 | 0.242 | 0.139 |
| Bacteria/Fibrobacteres | 0.006 MM | 0.138 | 0.702 |
| Bacteria/Firmicutes | 0.898 | 0.346 | 0.031 OS |
| Bacteria/Gemmatimonadetes | 0.003 MM | 0.162 | 0.789 |
| Bacteria/Kiritimatiellaeota | 0.059 | 0.341 | NA |
| Bacteria/Latescibacteria | 0.136 | 0.876 | 0.309 |
| Bacteria/LCP-89 | 0.234 | 0.576 | 0.837 |
| Bacteria/Lentisphaerae | 0.037 MM | 0.653 | 0.04 TM |
| Bacteria/Margulisbacteria | 0.228 | 0.546 | NA |
| Bacteria/Marinimicrobia_SAR406_clade | 0.048 MM | 0.091 | 0.191 |
| Bacteria/Nitrospirae | 0.538 | 0.146 | 0.212 |
| Bacteria/Omnitrophicaeota | 0.632 | 0.268 | 0.08 |
| Bacteria/Patescibacteria | 0.062 | 0.111 | 0.045 OS |
| Bacteria/Planctomycetes | 0.004 MM | 0.059 | 0.159 |
| Bacteria/Poribacteria | 0.087 | 0.39 | NA |
| Bacteria/Proteobacteria/Alphaproteobacteria | 0.738 | 0.000 TM | 0.000 TM |
| Bacteria/Proteobacteria/Deltaproteobacteria | 0.309 | 0.642 | 0.878 |
| Bacteria/Proteobacteria/Gammaproteobacteria | 0.006 MM | 0.375 | 0.001 TM |
| Bacteria/Rokubacteria | 0.412 | 0.682 | NA |
| Bacteria/Spirochaetes | 0.219 | 0.034 TM | 0.000 TM |
| Bacteria/Synergistetes | 0.687 | 0.595 | 0.302 |
| Bacteria/TA06 | 0.001 OS | 0.959 | 0.374 |
| Bacteria/Tenericutes | 0.34 | 0.312 | 0.023 OS |
| Bacteria/Verrucomicrobia | 0.027 MM | 0.648 | 0.072 |
| Bacteria/WOR-1 | 0.036 MM | 0.059 | 0.196 |
| Bacteria/WPS-2 | 0.694 | 0.543 | 0.484 |
| Bacteria/WS2 | 0.286 | 0.324 | 0.358 |
| Bacteria/Zixibacteria | 0.234 | 0.000 TM | 0.000 TM |
| Unclassified | 0.000 OS | 0.784 | 0.209 |
